# Correlation of neuter status and expression of heritable disorders

**DOI:** 10.1186/s40575-017-0044-6

**Published:** 2017-05-26

**Authors:** Janelle M. Belanger, Thomas P. Bellumori, Danika L. Bannasch, Thomas R. Famula, Anita M. Oberbauer

**Affiliations:** 1 Department of Animal Science, University of California, One Shields Ave, Davis, CA 95616 USA; 2 Department of Population Health & Reproduction, School of Veterinary Medicine, University of California, Davis, CA 95616 USA

**Keywords:** Neuter, Dog, Inherited disease

## Abstract

**Background:**

Gonadectomy, or neutering, is a very common surgery for dogs having many positive effects on behavior, health, and longevity. There are also certain risks associated with neutering including the development of orthopedic conditions, cognitive decline, and a predisposition to some neoplasias. This study was designed specifically to identify if a correlation exists between neuter status and inherited conditions in a large aggregate cohort of dogs representing many different breeds.

**Results:**

Neutered dogs were at less risk for early and congenital conditions (aortic stenosis, early onset cataracts, mitral valve disease, patent ductus arteriosus, portosystemic shunt, and ventricular septal defect) than intact dogs. Neutering was also associated with reduced risk of dilated cardiomyopathy and gastric dilatation volvulus in males. Neutering was significantly associated with an increased risk for males and females for cancers (hemangiosarcoma, hyperadrenocorticism, lymphoma, mast cell tumor, and osteosarcoma), ruptured anterior cruciate ligament and epilepsy. Intervertebral disk disease was associated with increased risk in females only. For elbow dysplasia, hip dysplasia, lens luxation, and patellar luxation neutering had no significant effect on the risk for those conditions. Neutering was associated with a reduced risk of vehicular injury, a condition chosen as a control.

**Conclusions:**

In this retrospective study, several conditions showed an increased risk associated with neutering whereas other conditions were less likely to be expressed in neutered dogs. The complexity of the interactions between neutering and inherited conditions underscores the need for reflective consultation between the client and the clinician when considering neutering. The convenience and advantages of neutering dogs that will not be included in a breeding program must be weighed against possible risk associated with neutering.

**Electronic supplementary material:**

The online version of this article (doi:10.1186/s40575-017-0044-6) contains supplementary material, which is available to authorized users.

## Plain English summary

Spaying and neutering of dogs is a well-accepted procedure in the United States and has many positive effects on behavior, health, and longevity. Although recent reports suggest that spaying and neutering may increase the occurrence of some joint disorders and some cancers, the relationship between inherited diseases and spay/neuter status has not fully been explored. The present study evaluated the prevalence and risk of genetic diseases, both early and late onset, in intact and neutered male and female dogs that were seen over a 15-year period at a university teaching hospital. Spayed and neutered dogs were at less risk for early and congenital conditions (aortic stenosis, early onset cataracts, mitral valve disease, patent ductus arteriosus, portosystemic shunt, and ventricular septal defect) than intact dogs. Neutered male dogs were at less risk for bloat (gastric dilatation volvulus) and dilated cardiomyopathy, whereas spayed females were at increased risk for intervertebral disk disease. Spaying or neutering in both sexes was significantly associated with an increased risk for cancers (hemangiosarcoma, hyperadrenocorticism, lymphoma, mast cell tumor, and osteosarcoma), ruptured anterior cruciate ligament, and epilepsy. For elbow dysplasia, hip dysplasia, lens luxation, and patellar luxation neutering had no significant effect on the risk for those conditions. A dog that was spayed or neutered was associated with a reduced risk of vehicular injury, a condition chosen as a control. The complexity of the interactions between spaying/neutering and inherited conditions underscores the need for reflective consultation between the client and the clinician when considering the procedure. The convenience and advantages of spaying or neutering dogs that will not be included in a breeding program must be weighed against possible risk associated with the procedure. Additionally, if owners elect to keep their dogs intact, they must then assume responsibility to vigilantly guard against unplanned litters.

## Background

Gonadectomy, or neutering, is a very common elective surgery in dogs. Current reports from the ASPCA of the United States indicate 83% of male and female dogs are neutered (http://www.aspca.org/animal-homelessness/shelter-intake-and-surrender/pet-statistics). Neutering has a number of benefits including eliminating unintended reproduction which in turn reduces the number of dogs that are unwanted and euthanized in shelters. Reproduction, pregnancy, and parturition are also associated with adverse health conditions such as sexually transmitted disease, pregnancy toxemia, metabolic disease, and dystocia [1, 2]. Reproductive disorders such as pyometra, mammary tumors, testicular cancer, ovarian cancer, and prostate cancer are also reduced/prevented with neutering (reviewed in [2]) although neutering has been associated with increased risk or aggressiveness of prostatic cancer [3–5]. Numerous studies evaluating the correlation of behavior with neutering have shown reduced aggression, mounting behavior, roaming, and urine marking [6, 7]. Neutering reduces the risk of biting in certain breeds of dogs [8, 9] and dogs that are neutered are at reduced risk for relinquishment [10]. Additionally, neutered dogs have been shown to have increased lifespan as compared to intact animals by 13.8 and 26.3% for males and females, respectively [11].

Studies have also shown that there are certain risks associated with neutering. Certain orthopedic conditions appear to be increased in neutered dogs [12–16]. Cognitive decline of aging dogs is accelerated in the neutered state [17] and neutering has been correlated with greater prevalence of immune disorders [18]. Despite the reduction in mammary and gonadal neoplasia, neutering is associated with increased prevalence of other cancers such as hemangiosarcoma, lymphosarcoma, mast cell tumor, and osteosarcoma [12, 13, 19–21].

The goal of this study was to assess if there exists a correlation between neuter status and diseases having a presumed inherited basis by evaluating a large cohort of dogs representing many different breeds. We hypothesized that for certain conditions, removing gonadal hormones by neutering would reduce the risk of disease expression whereas for other conditions, neutering would be associated with an increased risk of expression. Appreciating the limitations of a retrospective study and that not all dog breeds would exhibit all the conditions under study, the cohort of dogs seen at a teaching hospital were analyzed in aggregate with breed analysis secondary.

## Methods

Medical records for 90,090 individual dogs seen at the University of California William T. Pritchard Teaching Hospital from 1995 through the end of 2010 (a 15 year period) were categorized using methodology as previously described [22]. Dogs were classified as having one of the disease conditions if a confirmed diagnosis based on symptoms and test results was indicated in the records; diagnoses described as “possible” or “suspect” were not classified as a case nor were they considered unaffected (Additional file 1: Table S1). The entire medical record for each dog seen was interrogated for the diseases evaluated. Breed, age, and whether the dog was intact, or neutered was also tabulated. The date of initial diagnosis, if possible to determine from the medical record, was recorded; dogs with known neutering dates within 150 days of an initial diagnosis were classified as intact at the time of diagnosis. For the majority of dogs, age of neuter was not discernible from the records thereby precluding an assessment of age of neuter effects.

The disease conditions assessed were chosen because they are considered to be inherited (Online Mendelian Inheritance in Animals (OMIA) website (http://omia.angis.org.au/home/ and [23, 24]), have a relative surety of diagnosis, impact quality of life, and present in a high enough prevalence to permit analyses. Some of the conditions can also be considered congenital and those were: aortic stenosis, mitral valve disease, patent ductus arteriosus (PDA), portosystemic shunt, and ventricular septal defect [25–27]. The ocular disorders were lens luxation and early onset cataracts (those that appear at age 6 or earlier) [28] and orthopedic disorders were elbow dysplasia, hip dysplasia, intervertebral disk disease (IVDD), patellar luxation, and ruptured anterior cruciate ligament (RACL). Cancers evaluated were hemangiosarcoma, lymphoma, mast cell tumor, and osteosarcoma [23]. Hyperadrenocorticism, which can be the result of either hyperplastic overgrowth of pituitary adrenocorticotropin cells or tumors of the adrenal cortex [29], was also evaluated. Other disorders included dilated cardiomyopathy, gastric dilatation volvulus (GDV), and epilepsy. Vehicular injuries were used as a non-inherited control condition. Age of reported diagnosis for intact and neutered males and females in each condition were compared using a Student’s *T*-test with significance set at *P* < 0.05.

Data were categorized as binary traits (case or control) for each condition. Overall prevalence was calculated for each condition and the conditions were further categorized into one of 157 breed groups as well as one of four sex classes: intact females (F), neutered females (NF), intact males (M), and neutered males (NM). The final set of breed groups encompassed 153 recognized by the American Kennel Club (AKC) and 4 groups representing various crossbreds, Pit bulls, the AKC Foundation Stock Service (FSS) breeds, and the AKC miscellaneous breeds. Using a binomial density, the differences in the probability of a condition by sex class and breed group was calculated. For a given condition with sex class *i* (where *i* = F, NF, M, NM) and breed group *j* (*j* = 1,2,3, …, 157) we assume that n_cases ij_ ~ Binomial (n_cases ij_ + n_controls ij_, p_ij_) where n_cases ij_ was the number of observed cases in sex group *i* and breed group *j* and n_controls ij_ was the number of unaffected dogs in sex group *i* and breed group *j* and where p_ij_ was the probability of disease in sex and breed group *ij*. For each condition, p_ij_ for all *i* and *j*, was estimated as well as the odds ratio (OR) across the two neuter states for females and for males [30]. That is, the odds ratios for females and males, pooled across breeds, were defined as OR_F_ = [p_NF_/(1- p_NF_)]/[p_F_/(1- p_F_)] and OR_M_ = [p_NM_/(1- p_NM_)]/[p_M_/(1- p_M_)], respectively.

Evaluating these probabilities and odds ratios was facilitated through a hierarchical Bayesian framework implemented through the public-domain software Stan [31], accessed through the public-domain language R [32]. Using a hierarchical model permitted stable estimates of p_ij_ and valid error intervals for p_ij_ and therefore more credible OR estimates [33]. Estimating p_ij_ was done using log(p_ij_/(1- p_ij_)) = sex_i_ + breed_j_, with *i* being F, NF, M, NM, *j* = 1,2, …, 157, sex_i_ was the sex effect for group *i* and breed_j_ was the impact of breed *j* on disease prevalence. In our hierarchical Bayesian analysis we assumed the prior densities for the parameters of the model to be sex_i_ ~ N(0, ${\sigma}_s^2$) and breed_j_ ~ N(0, ${\sigma}_b^2$). Because these conditions were relatively uncommon, and some of the breed groups relatively small, the weakly informative prior of a half-Cauchy for ${\sigma}_s^2$ and ${\sigma}_b^2$ in our hierarchical model can bring some valuable stability to our estimates of p_ij_ and the subsequent OR values [34]. Specifically, we assume that ${\sigma}_s^2$ ~ Cauchy(0,5) and ${\sigma}_b^2$ ~ Cauchy(0,5), with both being limited to positive values. Evaluation of this hierarchical model was made possible with a Monte Carlo Markov Chain (MCMC) sampling process run in four chains. Each chain was based upon 20,000 total samples with the first 5,000 discarded as part of the warm-up process and the remainder thinned to every 20-th sample, resulting in a MCMC sample of 3,000 values [31]. The convergence to the posterior density was evaluated by the Gelman-Rubin test statistic and values below 1.05 indicate the adequacy of the MCMC sampling process for the data evaluated [35].

Having included breed in our analytic model, we were also able to evaluate the OR across the sex classes within each identified breed group. Accordingly, the ratios for males and females within breed group *j* are OR_Fj_ = [p_NFj_/(1- p_NFj_)]/[p_Fj_/(1- p_Fj_)] and OR_Mj_ = [p_NMj_/(1- p_NMj_)]/[p_Mj_/(1- p_Mj_)], respectively, for all groups *j* = 1, 2, …, 157. Like any OR, values that differ from 1.0 are considered evidence of inequality of disease risk across population subgroups. To evaluate the risk of neutering on disease prevalence we took advantage of the MCMC sampling process to compute the posterior probability of the OR exceeding 2.0. Choosing 2.0 rather than 1.0 permits a more cautious evaluation of the increase in disease risk that may be incurred from neutering. Being a probability, this value is bounded by the interval [0,1] and thus can be clearly represented in a heat map to identify trends in disease risk across breed groups.

To evaluate the risk of neutering on disease prevalence within a breed or group we took advantage of the MCMC sampling process to compute the posterior probability of the OR exceeding 1.0. Being a probability, this value is bounded by the interval [0,1] and thus can be clearly represented in a heat map to identify trends in disease risk across breed groups. To provide visual clarity to the heat map of disease prevalence, we divided the within breed posterior probabilities into one of five groups. Although a posterior probability is not equivalent directly to a hypothesis test, we consider here a general correspondence. That is, a posterior probability less than 0.05 implies that 95% of the time, the computed OR for a given within breed neuter status comparison was less than 1.0. Such an outcome is a strong indication that neutering significantly reduces the prevalence of disease. Similarly, a posterior probability greater than 0.95 implies that less than 5% of the computed OR values were less than 1, providing strong evidence that neutering increases the prevalence of disease in this breed group. Expanding upon this simple interpretation of the posterior probability of an OR > 1 we classified breeds groups, within a disease, into one of five groups: I. Posterior probability less than 0.05, strong indication that neutering reduces disease prevalence; II. Posterior probability between 0.05 and 0.10, evidence suggesting that neutering can reduce disease prevalence; III. Posterior probability between 0.10 and 0.90, no convincing evidence that neutering impacts disease prevalence; IV. Posterior probability between 0.90 and 0.95, evidence suggesting that neutering can increase disease prevalence; and V. Posterior probability greater than 0.95, strong indication that neutering increases disease prevalence.

## Results

In the study population of 90,090 dogs, 9,133 were F, 36,574 were NF, 12,555 were M, and 31,838 were NM. All the conditions assessed were less than 5% of the overall dog population studied with the exception of IVDD which was diagnosed in nearly 6% of the dogs seen at the hospital (Table 1). Ventricular septal defect was the lowest at 0.17%. Male and female condition distribution, without consideration of neuter status, was ~50% overall with some conditions being disproportionately higher in one sex vs. the other: PDA was higher in females and dilated cardiomyopathy and GDV higher in males (Table 1). Congenital conditions had the highest proportion recorded for intact dogs and for aortic stenosis, PDA, and ventricular septal defect in particular, intact dogs accounted for more than 60% of the cases.

**Table 1:** Demographic data for the conditions under study

Disease	% in Total Study Population	Females (n)	Neutered Females (n)	Males (n)	Neutered Males (n)	Ratio of Intact to Neutered	Ratio of Female to Male Prevalence
Congenital							
Aortic Stenosis	0.49	134	88	144	77	278 : 165	50.1 : 49.9
Mitral Valve Dysplasia	0.22	44	57	47	49	91 : 106	51.3 : 48.7
Patent ductus arteriosus	0.51	258	73	102	25	360 : 98	72.3 : 27.7
Portosystemic Shunt	0.74	161	172	158	178	319 : 350	49.8 : 50.2
Ventricular Septal Defect	0.17	63	25	53	16	116 : 41	56.1 : 43.9
Ocular							
Early onset Cataracts	0.88	152	241	151	246	303 : 487	49.7 : 50.3
Lens Luxation	0.44	39	178	41	138	80 : 316	54.8 : 45.2
Orthopedic							
Elbow Dysplasia	1.51	119	431	252	561	371 : 992	40.4 : 59.7
Hip Dysplasia	2.03	187	682	286	673	473 : 1355	47.5 : 52.5
Intervertebral disc disease	5.93	307	2047	819	2168	1126 : 4215	44.1 : 55.9
Patellar Luxation	2.75	284	1095	324	773	608 : 1868	55.7 : 44.3
Ruptured Anterior Cruciate Ligament	1.46	55	693	83	487	138 : 1180	56.7 : 43.3
Cancers							
Hemangiosarcoma	0.76	22	289	83	288	105 : 577	45.6 : 54.4
Hyperadrenocorticism (cushings)	1.39	39	704	84	424	123 : 1128	59.4 : 40.6
Lymphoma	1.9	81	733	222	672	303 : 1405	47.7 : 52.3
Mast Cell Tumor	1.82	76	844	175	549	251 : 1393	56.0 : 44.0
Osteosarcoma	0.89	33	343	84	341	117 : 684	46.9 : 53.1
Other							
Dilated Cardiomyopathy	0.44	23	116	94	162	117 : 278	35.2 : 64.8
Gastric Dilatation (Bloat)	0.28	25	73	60	93	85 : 166	39.0 : 61.0
Epilepsy	0.87	45	301	105	329	150 : 630	44.4 : 55.6
Vehicular Injury	2.44	328	680	509	683	837 : 1363	45.8 : 54.2

**Fig. 1:**
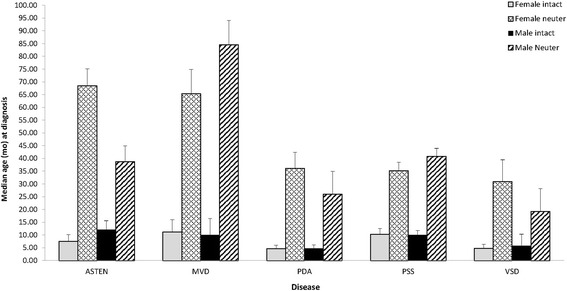
Median age at reported diagnosis for the congenital conditions of aortic stenosis (ASTEN), mitral valve disease (MVD), patent ductus arteriosus (PDA), portosystemic shunt (PSS), and ventricular septal defect (VSD). Female intact are *light gray bars*, neutered female are *light gray hatched bars*, male intact are *solid black bars*, neutered males *dark black hatched bars*

**Fig. 2:**
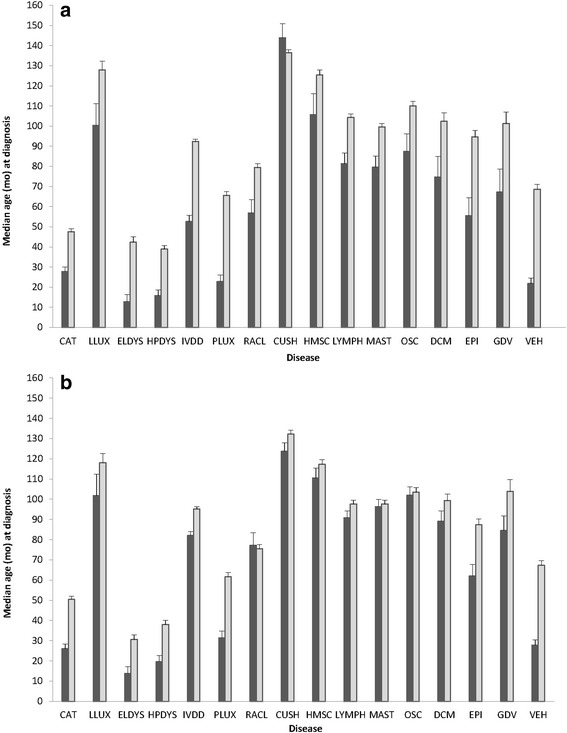
Median age (and standard error) at reported diagnosis for genetic conditions and vehicular injury for intact (*dark bars*) and neutered (*light bars*) females (**a**) and males (**b**). Conditions assessed were cataracts (CAT), lens luxation (LLUX), elbow dysplasia (ELDYS), hip dysplasia (HPDYS), intervertebral disk disease (IVDD), patellar luxation (PLUX), ruptured anterior cruciate ligament (RACL), hyperadrenocorticism (CUSH), hemangiosarcoma (HMSC), lymphoma (LYMPH), mast cell tumor (MAST), osteosarcoma (OSC), dilated cardiomyopathy (DCM), epilepsy (EPI), gastric dilatation volvulus (GDV), and vehicular injury (VEH)

The median age of neuter in the population for which age at neuter was known (*n* = 6,281) was 13.4 and 13.1 months for males and females, respectively [18]. The median age of neutering associated with early onset or congenital diseases was 7.54 ± 1.56 months for both males and females based upon known neuter ages of 262 dogs with these conditions (Fig. 1). For later onset inherited conditions, median age of neuter differed (*p* < 0.05) with females at 16.2 months (*n* = 371) and males at 22.8 months (*n* = 461). Intact dogs of both sexes tended to be diagnosed at earlier ages than neutered dogs for the majority of disorders (Fig. 2). Hyperadrenocorticism was the sole condition in which intact females and neutered females were diagnosed at statistically equivalent ages (P > 0.05). For males, dilated cardiomyopathy, GDV, lens luxation, RACL, hyperadrenocorticism, hemangiosarcoma, lymphoma, mast cell tumor, and osteosarcoma were diagnosed at ages that did not statistically differ for intact and neutered dogs.

The risk of all congenital conditions assessed was greater for the intact dogs of both sexes as seen in Table 2. Intact females were at 2.86 to 14 times higher risk than neutered females and intact males were at 2.3 to 10 times higher risk than neutered males for these early onset conditions. For the other conditions, intact dogs were at greater risk (~50% increased risk) for early onset cataracts and being involved in vehicular injuries. Intact males were at greater risk (~40-50% increased risk) for dilated cardiomyopathy and GDV and neuter status was not associated with lens luxation, elbow or hip dysplasia, IVDD, or patellar luxation. For females, there was no significant association of neuter status with disease expression for dilated cardiomyopathy, GDV, lens luxation, elbow or hip dysplasia, or patellar luxation and neutering was associated with a 70% increased risk of IVDD. For the cancers, epilepsy, and RACL, neuter status was positively associated with disease risk; males exhibited less of a risk associated with neutering than females.

**Table 2:**
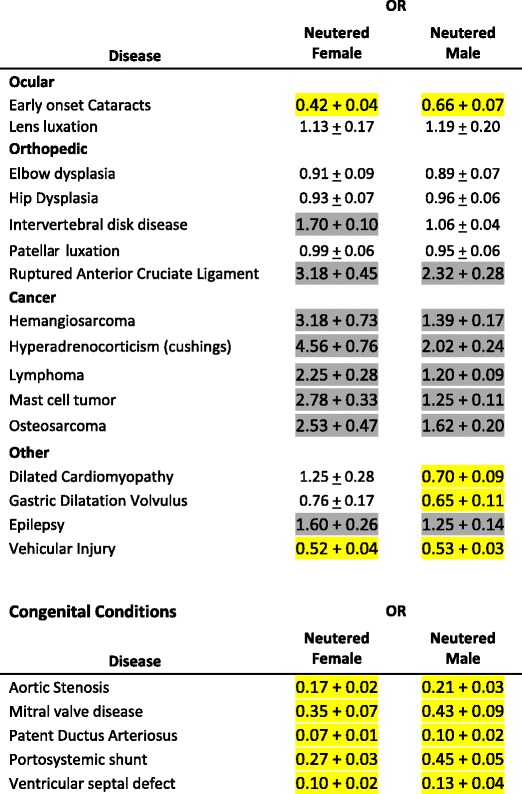
The odds ratio (OR) (± standard error) for the neutered dog relative to the intact dog for the conditions under study

Yellow shading indicates neutering was associated with reduced risk and dark gray shading indicates neutering was associated with an increased risk (*p* < 0.05). No shading indicates no difference in risk

The impacts of neutering within breeds was assessed, although extreme caution must be applied due to the small number of cases for many of the breeds (Additional file 1: Table S1). Nevertheless, inspecting the risk associated with neutering by breed may be informative (Additional file 2: Figures S1 and S2). The heat maps of risk, presented by AKC groups, reinforced the findings of the overall population, that is, when individual breeds were not considered. Exceptions in the overall population trend were seen in some breeds that showed either a reduction or enhancement of risk with neutering that differed from the aggregate risk, but upon closer inspection, the data were generally sparse for breeds showing a deviation from the overall trend. For example, osteosarcoma in breeds that showed reduced risk with neutering (Neopolitan mastiff, Airedale terrier, Bichon Frise, Basset hound, Bloodhound, Afghan hound, and Borzoi) all had zero cases in the NM sex class and one case in the M sex class which would correspond to a reduced risk with neutering. In conditions where there was no detected risk in the overall population certain breeds showed a reduced risk while others exhibited a greater risk. For example, the associated neuter risk with hip and elbow dysplasia varied noticeably across breeds. Females and/or males of the Belgian Malinois, Bouvier des Flandres, Collie, Old English sheepdog, Puli, and Shetland sheepdog herding breeds presented different hip dysplasia responses from the aggregate population. Yet those breeds each had a sex class with zero cases. In contrast, German shepherd dogs with large numbers of cases in each sex class showed no association between neutering and risk for hip dysplasia consistent with the aggregate population.

The category of mixed breed presented an interesting scenario. For the congenital, IVDD, RACL, GDV, cancers, and vehicular injury conditions females and males of the mixed breed category followed the risk associations seen for the population as a whole. For elbow dysplasia, hip dysplasia, patellar luxation, DCM, early onset cataracts, and lens luxation there were differences in risk for the mixed breeds from that seen in the general population that represented either an increase in risk for neutered females (elbow and hip dysplasia, DCM, and cataracts) or a decrease risk in neutered males (patellar luxation). Neutered mixed breed females were at less risk for lens luxation than that seen for the general population.

## Discussion

Numerous recent reports have centered on the advisability of neutering and unintended consequences associated with that surgical procedure [12–14, 19, 36, 37]. In this study, the goal was to assess the association of gonadal hormones on the expression of various conditions presumed to be inherited by assessing risk of expression associated with neutering. Knowledge of the interplay of neutering with certain conditions may inform medical decisions related to neutering. The findings of the present work, a retrospective association study, are consistent with some conclusions drawn in published literature. Specifically, our results indicate that for some inherited conditions, neutering provides a benefit and is associated with reduced risk whereas for other conditions the risk was elevated in the neutered dog, most especially the cancers and RACL.

As for any retrospective study, one must be mindful of the limitations presented in the data. The hospital whose records were interrogated, serves as a referral hospital in addition to being the primary care clinic for a limited geographical region. The data therefore may be biased toward a subset of dogs and may not reflect the dog population risk for disease associated with neutering. The congruence of the risk found in the present study with risk reported in past studies would suggest that this bias is not prohibitive. The absence of recorded age of neuter for the majority of the patients precluded associating a cause and effect premise between neuter and disease expression or inclusion of weight changes following the neuter. A prospective study of enormous proportions comprised of numerous breeds and sufficient numbers of each sex class would be necessary to confirm the risks identified in the present study. However, without that type of prospective study, defining the cause and effect of neutering on the expression of all the conditions outlined here is impossible and therefore one must rely upon associations and retrospective analyses and the recognition that association is not a demonstration of causality.

For all the conditions assessed, the congenital conditions of aortic stenosis, PDA, and ventral septal defect were the only conditions in which intact dogs exhibited a greater prevalence than neutered dogs. Although not significantly so, mitral valve dysplasia and portosystemic shunt had relatively high prevalence in intact dogs compared to the prevalence seen for all other conditions. Congenital conditions affect a dog’s quality of life and seemed to be diagnosed at relatively early ages with the dogs neutered soon thereafter likely accounting for the higher risk in intact dogs.

An increased risk associated with neutering for orthopedic conditions and cancers has reported previously for specific breeds [12–14]. For orthopedic conditions, typically the association is seen when early to late neutering is compared [12, 13, 16] with a predisposition of neutered dogs to exhibit joint disorders, especially in dogs neutered before sexual maturity [12, 13, 19, 20]. The association may be lost if the age of neuter is not accounted for as seen in the present study or the risk may be isolated to different breeds. A recent retrospective study by Hart et al. [14] highlighted the impact of breed on the risk associated with neutering in the expression of joint disorders; our breed data mirrored their findings that targeted Labrador and Golden retrievers. Terrier breeds tended to a greater risk with neutering whereas for nonsporting breeds, neutering was associated with a reduced risk. It is well understood that past sire and dam selection decisions within a breed alters gene frequency; disease expression patterns will be influenced by the interplay of altered gonadal hormones and the genetic background of the dogs [38, 39]. It must again be emphasized that low sample numbers of cases in each sex class requires caution in interpretation for individual breeds. The absence of significant risk observed for hip and elbow dysplasia and patellar luxation as reported in the present study for the aggregate dog population reflects that variable response in different breeds and in the variable statistical power based upon the number of cases for breeds.

In contrast to the above orthopedic conditions, cruciate ligament rupture is reported as a generalized risk in all neutered dogs [16, 40] a finding supported by the present study in which neutering of both sexes was associated with increased risk of RACL across breeds. The evidence for a protective role of sex hormones in ligament integrity can be seen in the presence of gonadal hormone receptors on ligaments [41], the increase of ligament elastin content and fiber diameter in response to estrogen administration [42], and the greater likelihood of cruciate injury in female athletes when estrogen is low [43]. However, obese dogs are four times more likely to rupture their cranial cruciate ligament [44] and neutering is a factor highly associated with the development of obesity, with neutered females being more prone than neutered males [45]. An obesity association, distinct from the gonadal hormone role, driving the observed increase in RACL cannot be discounted.

Of the genetic conditions evaluated in this study, IVDD was the most prevalent, a finding consistent with a previous retrospective study [46]. Previous studies also report that intact males have a one to two-fold higher IVDD prevalence than intact females [46–48]. As reviewed by Brisson [49], some studies report males and neutered females are at higher risk for IVDD. The elevated risk of IVDD in neutered females is also consistent with a recent study in rats that demonstrated ossification of the intervertebral disk endplate, which provides nutrients to the disk through marrow contact channels, in response to ovariectomy [50]. Upon estrogen supplementation of the ovariectomized animals, the authors observed an increase in channel volume, improved nutrient flow to the disk, and reduced IVDD progression. Similar impairment of vertebral bone structure is observed in post-menopausal women. As estrogen levels decline with menopause, bone structure is compromised, notably with intervertebral disk damage (as reviewed by [51–53]). Removing the positive effects of estrogen on bone by neutering female dogs would be expected to increase their risk for IVDD compared to intact females.

A presumed protective role of estrogen was seen in many of the assessed conditions, notably cancer. Cancer risk has been associated with neuter status in the literature [11, 54, 55]. In the present study, age of diagnosis for neutered and intact males was statistically equivalent for most cancers with the exception of lymphoma. In contrast, age of cancer diagnosis was statistically later for neutered females for all cancers except hyperadrenocorticism. This sex discrepancy of age at diagnosis may account for the greater risk observed for neutered females than neutered males. A greater risk for cancers associated with neutering may reflect the (non-significant) tendency for neutered dogs to be older when diagnosed and the greater longevity in neutered dogs [11, 56, 57] as advancing age is associated with a greater prevalence of cancers [24, 58].

Importantly, however, is that not all cancers in dogs show an increased risk with neutering. The incidence of mammary cancer is virtually eliminated if females are neutered prior to sexual maturity [59] whereas the frequency of other cancers, such as melanoma and squamous cell carcinoma, are not influenced by neuter status [11]. What accounts for the susceptibility to cancer upon removal of gonadal steroids is unclear. An additional confounding effect is the role of neutering on the development of obesity and the association with joint disorders and increased incidence of cancers with obesity [60, 61]. The elevated risk of the conditions seen in the present study, including the cancers, may be a secondary effect of increased body weight and altered metabolism [62, 63].

Interestingly, the risk of epilepsy was higher in the neutered population, coinciding with a report on dogs in the United Kingdom [64]. Gonadal hormones, most especially estrogen, has been shown to reduce the seizure threshold cells in both humans and laboratory animals whereas progesterone is considered to be “anticonvulsant” (reviewed in [65, 66]. Despite those generalizations, estrogen has differential effects in animal models of epilepsy with it being promotional in some induced models and anticonvulsant in others [65]. Estrogens have also been suggested to be anticonvulsant when doses are physiological and not supra-physiological [67]. Progesterone on the other hand reduces the likelihood of seizures. The intact female dog has elevated progesterone for some months during its reproductive cycle which may account for a lowered risk for intact females. The role of testosterone in seizure response has been less well studied [67] and the risk seen in neutered males was relatively low compared to the intact males perhaps reflecting the conversion of endogenous testosterone to a protective estradiol form.

Retrospective studies of dogs have demonstrated breed predispositions in cataract prevalence with equivalent male female distribution, though neuter status was not recorded [28, 68]. The cataracts analyzed in the present study displayed greater risk for intact dogs of both sexes with females showing a higher risk than males with a similar breed predisposition as reported in the above mentioned studies. The female sex hormones have been implicated in the development of lens opacities and cataracts [69] based upon observations that women develop cataracts more frequently than men, a greater cataract incidence occurs in post-menopausal women, and exogenous estrogen plays a protective role in ovariectomized rats [70]. However a study by Colitz et al., [71] showed that cataractous lens epithelial cells overexpress estrogen receptors and the authors posited that these cells may become estrogen sensitive during caractogenesis. Furthermore, lens epithelia have the aromatase pathway enabling conversion of testosterone to estradiol [72]. This would indicate a greater risk associated with intact dogs of both sexes.

The male preponderance for dilated cardiomyopathy [73] was seen in the present data with males accounting for 64.8% of the cases. Neutered males, but not females, had a reduced risk for dilated cardiomyopathy strongly suggesting a permissive effect of testosterone on expression of this disease, a supposition supported by the anabolic steroid literature. Administration of anabolic steroids to dogs results in hypertrophy of cardiac muscle [74] mimicking the cardiomyopathy conditions seen in anabolic steroid use in athletes [75].

The overall prevalence of GDV being greater in males than females agrees with published findings [76]. Although the present study indicates that neutering reduced the risk of GDV, significantly so in males, other published studies report variable impact of sex and neuter status ([77] and reviewed in [78]). The inconsistent association of neuter status on risk of GDV likely reflects the complex nature of the condition with the sex hormones being just one of the many factors that influence expression. Neutering was highly associated with increased risk within the breeds of the AKC working group; however, those breeds tended to have the highest prevalence of GDV.

The risk of vehicular injuries was also reduced in the neutered dogs possibly due to associated differences in owner behavior and/or dog behavior. A study on owner reported trainability indicated that for some breeds neutering improved trainability though the authors caution against broad generalization across breeds arguing more for breed specific assessment [79]. Dogs that have not been trained or do not respond to an owner’s command may be more likely to enter traffic. Neutering has been associated with alterations in dog behavior, reducing the risk of biting in certain breeds of dogs [8] and neutered dogs are at reduced risk for relinquishment presumably due to improved behavior [10]. A study that evaluated boldness in dogs found that intact dogs of both sexes and multiple breeds are more “bold” than neutered dogs [80] which may be associated with exploratory and wandering behavior. Additionally, neutered dogs are less aggressive and less likely to roam [6, 7]. These studies suggest that the impact of neutering on dog behavior could account for the reduced risk of vehicular injury.

Despite the limited ability to assess individual breed contribution to the risk associated with neutering, several key points can be drawn. The breeds making up the herding, non-sporting, sporting, and working AKC groups were more likely to display an association with neutering for risk of certain conditions. Also, the mixed breed group, which reflects an amalgamation of various breeds, would be expected to reflect the overall population risk response, which was for the most part observed, validating the approach of determining an overall dog population response. Finally, neutering in females presented a generally greater risk for the conditions than that detected in males.

## Conclusions

Risk assessment based upon retrospective studies should be applied to clinical settings and client recommendations with caution because of the limitations of such studies. In the present study, factors such as age of neuter, breed, and concomitant metabolic conditions such as obesity were not incorporated into the analyses. Yet the fact of a neutering risk that exists for certain conditions, most especially in females, cannot be ignored and underscores the need for reflective consultation between the client and the clinician when considering neutering. For dogs not to be used in planned breedings, the convenience and advantages of neutering dogs must be weighed against possible risks associated with neutering.

## Additional files

Additional file 1: Table S1. Number of cases and controls for each condition by breed used in the study. (XLSX 48 kb)

Additional file 2: Figures S1 and S2. Heat map of risk associated with neutering in females (Figure S1) and males (Figure S2) by dog breed, assembled into AKC breed groupings. Heat map represents classification of one of five categories: I. Posterior probability less than 0.05, strong indication that neutering reduces disease prevalence (green); II. Posterior probability between 0.05 and 0.10, evidence suggesting that neutering can reduce disease prevalence (light green/teal); III. Posterior probability between 0.10 and 0.90, no convincing evidence that neutering impacts disease prevalence (blue); IV. Posterior probability between 0.90 and 0.95, evidence suggesting that neutering can increase disease prevalence (peach); and V. Posterior probability greater than 0.95, strong indication that neutering increases disease prevalence (red). Refer to Additional file 1: Table S1 for breed names associated with breed codes. (ZIP 7915 kb)

## Abbreviations

AKC: American Kennel Club
DCM: Dilated cardiomyopathy
F: Intact Female
FSS: Foundation Stock Service
GDV: Gastric dilatation volvulus
IVDD: Intervertebral disk disease
M: Intact male
NF: Neutered female
NM: Neutered Male
OR: Odds Ratio
PDA: Patent ductus arteriosus
RACL: Ruptured anterior cruciate ligament
