# Supplementary figures and images for: Correlation of neuter status and expression of heritable disorders
Source: Canine Genet Epidemiol. 2017 May 26;4:6. doi: 10.1186/s40575-017-0044-6 (PMC5445488; doi:10.1186/s40575-017-0044-6)

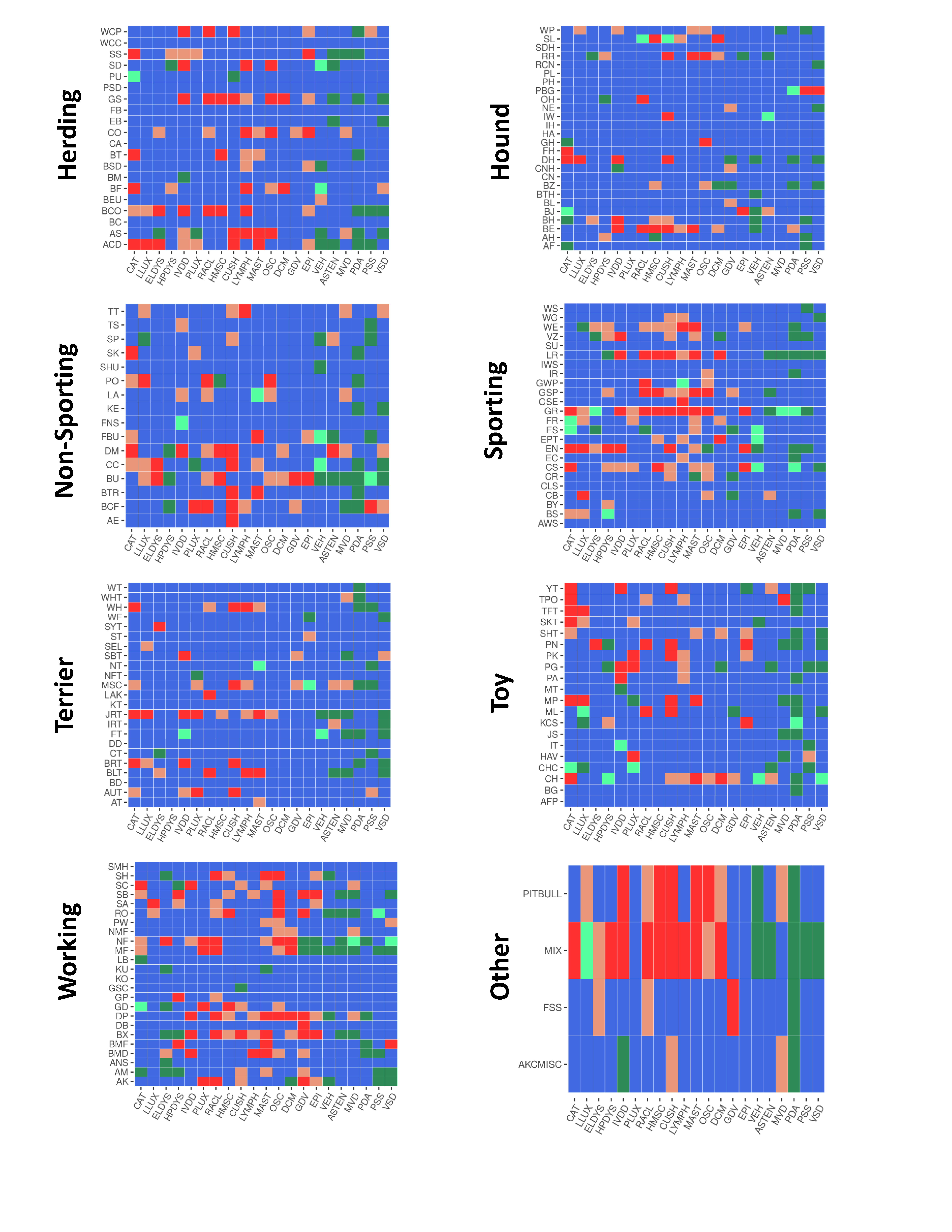

Supplement: Supplementary file 2 — Heat map of risk associated with neutering in females (Figure S1) and males (Figure S2) by dog breed, assembled into AKC breed groupings. Heat map represents classification of one of five categories: I. Posterior probability less than 0.05, strong indication that neutering reduces disease prevalence (green); II. Posterior probability between 0.05 and 0.10, evidence suggesting that neutering can reduce disease prevalence (light green/teal); III. Posterior probability between 0.10 and 0.90, no convincing evidence that neutering impacts disease prevalence (blue); IV. Posterior probability between 0.90 and 0.95, evidence suggesting that neutering can increase disease prevalence (peach); and V. Posterior probability greater than 0.95, strong indication that neutering increases disease prevalence (red). Refer to Additional file 1: Table S1 for breed names associated with breed codes. (ZIP 7915 kb) [file 40575_2017_44_MOESM2_ESM.zip › Supplemental Figure 1.tif]

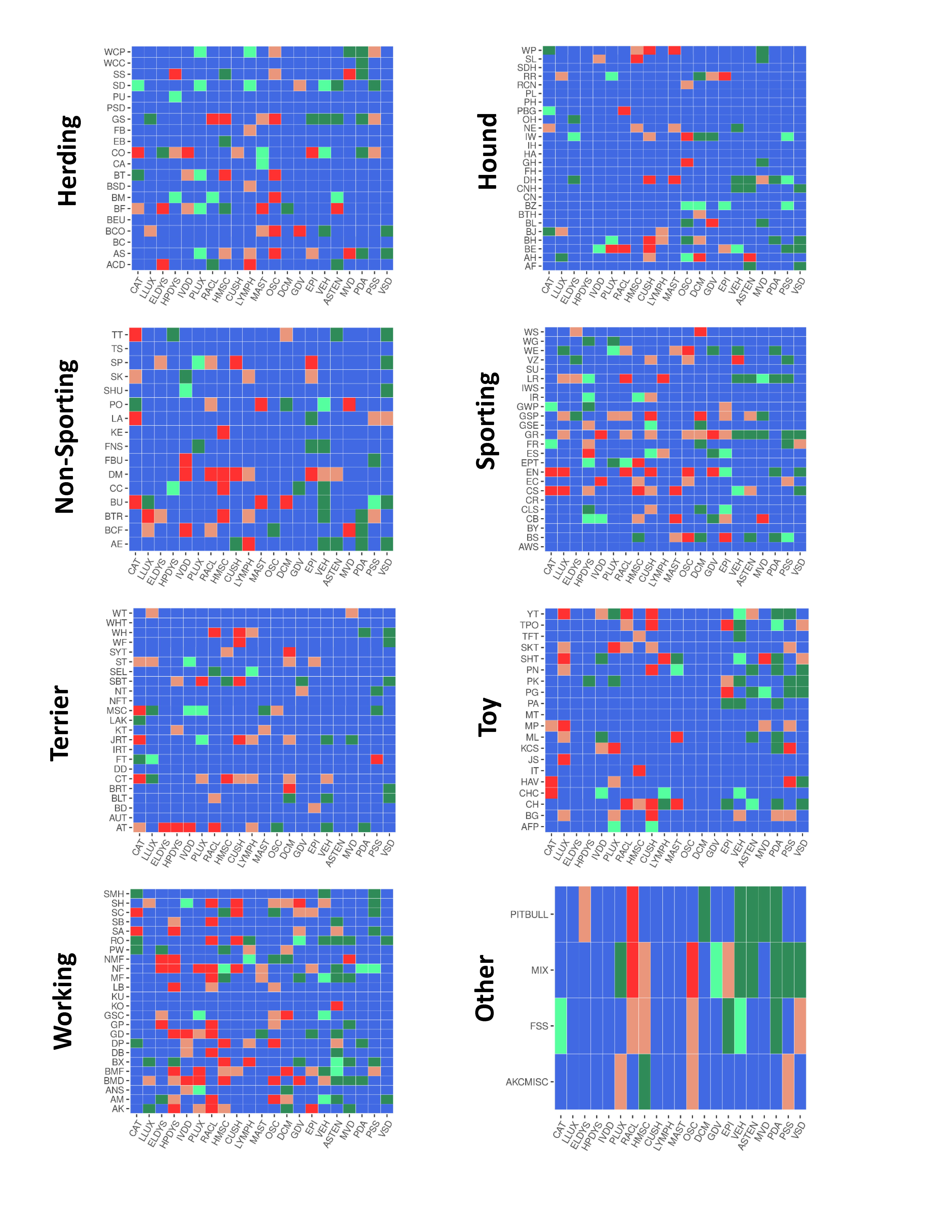

Supplement: Supplementary file 2 — Heat map of risk associated with neutering in females (Figure S1) and males (Figure S2) by dog breed, assembled into AKC breed groupings. Heat map represents classification of one of five categories: I. Posterior probability less than 0.05, strong indication that neutering reduces disease prevalence (green); II. Posterior probability between 0.05 and 0.10, evidence suggesting that neutering can reduce disease prevalence (light green/teal); III. Posterior probability between 0.10 and 0.90, no convincing evidence that neutering impacts disease prevalence (blue); IV. Posterior probability between 0.90 and 0.95, evidence suggesting that neutering can increase disease prevalence (peach); and V. Posterior probability greater than 0.95, strong indication that neutering increases disease prevalence (red). Refer to Additional file 1: Table S1 for breed names associated with breed codes. (ZIP 7915 kb) [file 40575_2017_44_MOESM2_ESM.zip › Supplemental Figure 2.tif]
